# Evaluation of chemotherapy-induced nausea and vomiting in pediatric patients with high-grade glioma treated with lomustine—a case series

**DOI:** 10.1007/s00520-024-08474-7

**Published:** 2024-04-16

**Authors:** Kim P. J. Schellekens, Sarah Babette Hageman, Els C. Haverkate, Marianne D. van de Wetering, Frederike K. Engels, Aeltsje Brinksma, Evelien de Vos-Kerkhof

**Affiliations:** 1 Princess Máxima Center for Pediatric Oncology, Utrecht, the Netherlands; 2 Department of Pediatric Oncology, Sophia Children’s Hospital, Erasmus Medical Center, Rotterdam, the Netherlands

**Keywords:** Lomustine, Chemotherapy-induced nausea and vomiting, CINV, Anti-emetics, Pediatric high-grade glioma

## Abstract

**Purpose:**

Although lomustine has been used as a chemotherapeutic agent for decades, no recommendation on appropriate chemotherapy-induced nausea and vomiting (CINV) prophylaxis is available. As CINV is considered one of the most bothersome side effects of chemotherapy, adequate prophylaxis is of relevance to improve quality of life during cancer treatment. The aim of this retrospective case series was to report the incidence and severity of CINV in pediatric patients with high-grade glioma treated with lomustine and to formulate recommendations for appropriate CINV prophylaxis.

**Methods:**

Pediatric patients treated with lomustine for high-grade glioma according to the ACNS 0423 protocol were identified retrospectively. Two researchers independently reviewed and classified complaints of CINV and administered CINV prophylaxis. Treatment details, tumor localization, and response to therapy were systematically extracted from the patients’ files.

**Results:**

Seventeen children aged 8–18 years received a median of four cycles of lomustine. CINV complaints and administered prophylaxis were evaluable in all patients. Moderate or severe CINV was observed in 13/17 (76%) patients. Administered prophylactic CINV regimens varied from no prophylaxis to triple-agent combinations.

**Conclusion:**

In this case series, we identified lomustine as a highly emetogenic chemotherapeutic agent. According to the current guidelines, CINV prophylaxis with a 5-HT3 receptor antagonist in combination with dexamethasone and (fos)aprepitant is recommended.

## Background

Lomustine, also known as CCNU (chloroethyl-cyclohexyl-nitrosourea), is an orally administered alkylating antineoplastic agent that was first authorized for use by the European Medicines Agency in 1978 [1]. It is currently approved for the treatment of brain tumors, small-cell lung cancer, malignant melanoma, Hodgkin’s disease, and non-Hodgkin lymphoma [1, 2]. Within the pediatric population, lomustine is most commonly used in the treatment of malignant brain tumors, including medulloblastoma and high-grade glioma. The addition of lomustine to adjuvant treatment with temozolomide significantly improved overall survival and event-free survival in pediatric high-grade glioma [3, 4].

Chemotherapy-induced nausea and vomiting (CINV) are among the most common side effects of cancer therapy and can have serious clinical consequences, including malnutrition, dehydration, acid–base, and electrolyte disturbances. In addition, CINV has been shown to negatively impact (health-related) quality of life and, in case of patients’ refusal to continue chemotherapy cycles, can compromise treatment efficacy [5–9]. As the emetogenicity of the chemotherapeutic agent is the most important determinant of the occurrence of CINV, current recommendations regarding prophylaxis are founded on this characteristic [10, 11].

In an evidence-based classification, the emetogenicity of frequently used chemotherapeutics is grouped into four categories, being: minimal, low, moderate, or high [12]. However, for lomustine, the emetogenic potential has not yet been described systematically, and thus, no evidence-based recommendations regarding the required prophylaxis have been formulated. The aim of this retrospective case series was to report the incidence and severity of CINV in pediatric patients with high-grade glioma treated with lomustine and to formulate recommendations for appropriate CINV prophylaxis.

## Methods

This retrospective case series includes children with high-grade glioma treated according to the ACNS 0423 protocol in the Princess Máxima Center for Pediatric Oncology in Utrecht, the Netherlands, between February 2018 and August 2023. Children were included when informed consent for the use of clinical data was obtained and at least one cycle of the ACNS 0423 treatment protocol including lomustine was administered. In the ACNS 0423 protocol, patients received a radiotherapy dose of 54.0 Gy in 30 fractions of 1.8 Gy, in combination with temozolomide 90 mg/m^2^/day for 6 weeks. Four weeks after radiotherapy was completed, adjuvant therapy with 6-week cycles of lomustine 90 mg/m^2^ on day 1 and temozolomide 160 mg/m^2^/day on days 1–5 were administered, for a maximum of six cycles [4]. This treatment protocol was selected because the emetogenicity of temozolomide is considered low, and therefore, the observed CINV complaints during these courses are considered primarily attributable to lomustine [12].

Pharmacy records were used to identify all possibly eligible patients. The use of CINV prophylaxis was classified into three categories: category 1 (low) consisting of a selective serotonin receptor (5-HT3) antagonist such as granisetron or ondansetron; category 2 (moderate) consisting of category 1 combined with dexamethasone; category 3 (high) consisting of category 2 combined with (fos)aprepitant. Granisetron was used as the 5-HT3 receptor antagonist of first choice for intravenous administration at the outpatient clinic (dosed 0.045 mg/kg/dose, twice daily), or ondansetron for oral administration at home (dosed 5 mg/m^2^/dose, three doses per day). In children ≥ 33 kg, aprepitant is dosed 125 mg once daily on day 1, and 80 mg once daily on days 2 and 3. Children < 33 kg received a weight-adjusted dose. Doses of rescue medication are discussed on an individual basis (Table 3).

Data on the following matters were extracted from electronic health records: demographic information, specifics about the disease (e.g., primary site) and treatment (e.g., dose modifications), use of CINV prophylaxis, the occurrence of tumor progression, and increased intracranial pressure. Notes regarding the following topics were analyzed to determine the severity of overall CINV: nausea, vomiting, (change in) appetite, nutritional intake, tube feeding, and weight loss. Based on these observations, two independent researchers scored CINV symptoms as follows: none ( −), mild (±), moderate (+), severe (+ +). In addition, patients’ primary care providers were asked to score the severity of CINV in retrospect. Ultimately, children were classified to have experienced none to mild (scores 0 and 1), moderate (scores 2 and 3), or severe (scores 4 and 5) CINV.

## Results

### Demographics

Between February 2018 and August 2023, 18 children with high-grade glioma were treated according to the ACNS 0423 protocol. Seventeen patients with a median age of 14 years (range 8–18) met the defined inclusion and exclusion criteria and were eligible for retrospective data collection. One patient was ineligible because no informed consent for the use of clinical data was obtained. Baseline characteristics are summarized in Table 1.

**Table 1:** Baseline characteristics

Patient characteristic	Frequency (%)	Median (range)
Sex		
Male	12 (70.6%)	
Female	5 (29.4%)	
Age (years)		14 (8–18)
Pathologic diagnosis (CNS WHO 2021 classification)		
Diffuse pediatric-type high-grade glioma, H3-wild-type and IDH-wild-type	12 (70.6%)	
Diffuse hemispheric glioma, H3 G34-mutant	4 (23.5%)	
Diffuse non-midline glioma, H3 K27M-mutant NEC	1 (5.9%)	
Primary site		
Hemispheric	15 (88.2%)	
Thalamus	1 (5.9%)	
Spinal	1 (5.9%)	
Metastatic disease		
Yes	7 (41.2%)	
No	10 (58.8%)	
First line treatment		
Yes	15 (88.2%)	
No	2 (11.8%)	

*H3* histone 3, *IDH* isocitrate dehydrogenase, *NEC* not elsewhere classified

### Treatment

A median number of four cycles (range 1–8) containing lomustine with (*n* = 13) or without (*n* = 4) temozolomide were administered. Lomustine doses were reduced in four patients, mainly due to hematological toxicity. Treatment details are summarized in Table 2.

**Table 2:** Treatment details

ID	Sex	Age	Completed cycles	Dose modifications lomustine per cycle	Temozolomide co-administered
1	M	13	2	(1, 75%; 2, 75%)	Yes
2	M	18	3	-	No
3	F	18	4	-	Yes
4	M	13	1	-	Yes
5	F	16	1	-	Yes
6	M	13	3	-	Yes
7	M	9	4	-	Yes
8	F	8	4	(3, 80%; 4, 80%)	Yes
9	M	14	4	-	No
10	F	16	6	-	Yes
11	M	15	4	-	No
12	M	15	7	-	Yes
13	M	10	2	-	Yes
14	M	9	5	(4, 75%; 5, 75%)	Yes
15	F	15	8	-	No
16	M	14	6	-	Yes
17	M	8	5	(3, 78%; 4, 50%; 5, not administered; 6, 50%)	Yes

### Chemotherapy-induced nausea and vomiting

Clinical data on CINV prophylaxis and symptoms were available for all patients (Table 3). Severe CINV, defined by a score of 4 or 5, was observed in 6/17 (35%) patients. In an additional 7/17 (41%) patients, moderate CINV was observed, defined by a score of 2 or 3. Hence, a total of 13/17 (76%) of patients experienced moderate or severe CINV.

**Table 3:** Administered CINV prophylaxis and observed CINV symptoms during treatment

ID	CINV prophylaxis during treatment*	CINV rescue medication	CINV symptoms during treatment**				CINV score***
			*Nausea*	*Vomiting*	*Appetite/intake*	*Weight loss*	
1	No		-	-	-	-	0
2	No		n.a.	n.a.	-	-	0
3	1		±	±	+	-	2
4	1		+ +	+ +	n.a.	n.a.	4
5	1		+	-	+	+	3
6	1	MCP 0.5 mg/kg/day	+ +	+ +	±	+	5
7	1		-	+	n.a.	-	1
8	1		+ +	+ +	±	+	5
9	1		+	-	-	-	1
10	1		+ +	+	-	-	3
11	1		+ +	+	+	-	4
12	2		±	±	±	±	2
13	3		+	±	±	+	3
14	3	MCP 0.6 mg/kg/day	+ +	+ +	+	-	5
15	3		+	+	+	+	4
16	3		+ +	-	-	-	2
17	3		-	±	±	+	2

*MCP* metoclopramide

*1, 5-HT3 receptor antagonist; 2, 5-HT3 receptor antagonist + dexamethasone; 3, 5-HT3 receptor antagonist + (fos)aprepitant +/- dexamethasone

**- none, ± mild, + moderate, +  + severe, *n.a.* not available

***CINV score 0 and 1, none to mild; 2 and 3, moderate; 4 and 5, severe. Based on CINV symptoms during treatment, CINV prophylaxis during treatment, and score of primary care provider

CINV prophylaxis was administered in 15/17 patients (88%), of whom 5/15 (33%) received a regimen including (fos)aprepitant. In one patient (#14), a CINV score of 5 was observed despite CINV prophylaxis with aprepitant, while no signs of CINV were observed in two patients (#1 and #2) receiving no anti-emetic prophylaxis. In the single patient with an extracranial tumor located in the spinal cord (#12), a CINV score of 2 was observed.

## Discussion

We present a retrospective case series of 17 children and adolescents treated for high-grade glioma with lomustine, in whom we evaluated CINV symptoms and prophylaxis to formulate a recommendation regarding anti-emetic prophylaxis. We observed moderate to severe CINV in 76% of children. Following the classification algorithm described by Paw Cho Sing et al., lomustine could be classified as a highly emetogenic chemotherapeutic agent [12]. According to current CINV prophylaxis guidelines, triple therapy with (fos)aprepitant, dexamethasone, and a 5-HT3 receptor antagonist is recommended [13].

The role of dexamethasone as an anti-emetic drug in brain tumors remains controversial [14]. In several treatment guidelines, its use is not recommended. In vitro studies have shown that administration of dexamethasone results in a reduced permeability of the blood–brain barrier, which may lead to a restricted penetration of chemotherapy to the brain and thus to a diminished effectiveness of chemotherapy in the central nervous system [15–17]. However, no robust clinical evidence supporting this explanation is available.

Data from previously conducted prospective clinical trials evaluating lomustine in children did not describe the occurrence of nausea and vomiting in sufficient detail for the purpose of this analysis [4, 18]. In the pediatric phase 1 dose-finding study of lomustine and temozolomide, nausea and vomiting were excluded from the definition of dose-limiting toxicity, and non-hematological toxicity was not reported in detail [18]. In the subsequent pediatric phase 2 study (ACNS 0423), grade 3 or 4 nausea was reported in 5.6% of patients. Grade 1 or 2 adverse events related to nausea and vomiting nor administered CINV prophylaxis were reported [4]. Hence, this data cannot be used in the algorithm described by Paw Cho Sing et al., which is based on the administered prophylaxis and the presence of CINV complaints, rather than the severity of the complaints (e.g., according to the CTCAE-grading) [12].

Our findings are in line with what is known for carmustine (BCNU; β-chloro-nitrosourea), another chemotherapeutic agent in the class of nitrosoureas. Although pediatric-specific data is sparse, carmustine has been classified as highly emetogenic in adults [11]. Our observations reflect the inter-individual heterogeneity in susceptibility to CINV. Interestingly, two patients did not experience CINV complaints while not receiving any anti-emetic prophylaxis. On the other hand, two other patients experienced severe CINV while receiving CINV prophylaxis according to highly emetogenic chemotherapy (HEC) guidelines. Although our observations indicate that lomustine should be considered a highly emetogenic chemotherapeutic agent, critical evaluation of alternative causes for nausea and vomiting remains essential. Especially in patients with high-grade brain tumors, in whom tumor progression can occur rapidly and can result in increased intracranial pressure. In the cases presented here, alternative causes for nausea and vomiting (e.g., tumor progression, tumor localization) were systematically evaluated and considered not to have interfered with the assessment of CINV.

Despite the fact that our analysis is solely based on observations in children and adolescents, our observations could be of relevance to the adult population. In the phase 3 trial evaluating lomustine and temozolomide in adult patients with glioblastoma, nausea was observed in 30% of patients and vomiting in 9% of patients [19]. However, extrapolation should be done with caution, due to the differences in the efficacy of CINV prophylaxis between children and adults. Complete response rates of HEC CINV prophylaxis have shown to be almost 20% lower in the pediatric population compared to the adult population [20, 21]. Possible explanations for this difference encompass intrinsic differences in the pathogenesis of CINV, differences in emetogenicity or administered doses of chemotherapy, adjusted use of or sensitivity to anti-emetic treatment, and differences in metabolic profiles [20]. In addition, a prospective pharmacokinetic interaction study was performed in children receiving intravenous dexamethasone and (fos)aprepitant. This study showed that (fos)aprepitant had less influence on dexamethasone clearance in children compared to what has been observed in the adult population, resulting in lower dexamethasone exposure in children compared to adults. The lower dexamethasone exposure in children could contribute to the poorer CINV control [22].

Inherent to the study design, a limitation of this case series is the lack of a control group which makes it difficult to state direct causal inferences. Furthermore, the retrospective approach carries a risk of observer bias, as we relied on the reported observations and interpretations of different healthcare providers. No validated CINV scores were collected prospectively. However, by having scored the CINV severity by the patients’ primary care provider in addition to two independent researchers, we pursued to reduce this risk of bias*.* The evaluated reports did not consequently contain sufficiently detailed information to distinguish between acute CINV (< 24 h after chemotherapy administration), and delayed CINV (24–72 h after chemotherapy administration), potentially explained by the outpatient setting in which these patients were treated. Therefore, the focus in this case series lies on the description of overall CINV related to lomustine administration.

Ideally, we would have preferred to evaluate the emetogenic potential of lomustine monotherapy. However, as single-agent lomustine is not commonly used in daily practice; this was not considered feasible. We selected patients treated according to the ACNS 0423 protocol, consisting of a combination of lomustine and temozolomide. In other treatment protocols (e.g., ACNS 0331 [23]), lomustine is co-administered with cisplatin, which by itself is considered highly emetogenic [12]. By selecting a protocol that combines lomustine with temozolomide, which is a chemotherapeutic agent that has been classified as low emetogenic, we aimed to conduct an evaluation that best reflects the emetogenicity of lomustine [12]. However, we cannot rule out individual differences in the sensitivity to adverse events of temozolomide. Lastly, we present a relatively small cohort of patients. Therefore, a subgroup analysis on the influence of potential confounders (e.g., gender, tumor localization, and/or co-administration of temozolomide) was not considered feasible within this case series.

In this retrospective case series, we classified lomustine as a highly emetogenic chemotherapeutic agent. Appropriate to this classification, current CINV prophylaxis guidelines recommend triple therapy with (fos)aprepitant, dexamethasone, and a 5-HT3 receptor antagonist [13]. Although lomustine has been used in cancer treatment for decades, no comprehensive evaluation of CINV symptoms and prophylaxis has been conducted to date. The occurrence and severity of CINV should be evaluated during the earlier phases of clinical development of novel therapeutics, so that well-founded recommendations regarding anti-emetic prophylaxis are available when treatments are introduced to clinical care. In addition, there is a high need for the implementation of validated methods to systematically evaluate CINV complaints in children. The availability of such an instrument would improve the standardized and uniform analysis and reporting of CINV complaints.

## Data Availability

No datasets were generated or analyzed during the current study.
